# Anthropogenic noise may impair the mating behaviour of the Shore Crab *Carcinus Maenas*

**DOI:** 10.1371/journal.pone.0276889

**Published:** 2022-10-27

**Authors:** Kara Rising, Jörg Hardege, Tom Tregenza, Martin Stevens

**Affiliations:** 1 Centre for Ecology and Conservation, College of Life & Environmental Sciences, University of Exeter, Penryn Campus, Penryn, United Kingdom; 2 Aquatic Research Facility, Environmental Sustainability Research Centre, University of Derby, Derby, United Kingdom; 3 Department of Biological and Marine Sciences, Hull University, Hull, United Kingdom; Wildlife Conservation Society Canada, CANADA

## Abstract

Anthropogenic noise is a recent addition to the list of human-made threats to the environment, with potential and established negative impacts on a wide range of animals. Despite their economic and ecological significance, few studies have considered the impact of anthropogenic noise on crustaceans, though past studies have shown that it can cause significant effects to crustacean physiology, anatomy, and behaviour. Mating behaviour in crustaceans could potentially be severely affected by anthropogenic noise, given that noise has been demonstrated to impact some crustacean’s ability to detect and respond to chemical, visual, and acoustic cues, all of which are vital in courtship rituals. To explore if noise has an impact on crustacean mating, we tested the responses of male green shore crabs (*Carcinus maenas*) from the southwest UK coast by exposing them to ship noise recordings while simultaneously presenting them with a dummy-female soaked in the female-sex pheromone uridine diphosphate (UDP) in an experimental tank setup (recording treatment: n = 15, control treatment: n = 15). We found a significant, negative effect of noise on the occurrence of mating behaviour compared to no noise conditions, though no significant effect of noise on the time it took for a crab to respond to the pheromone. Such effects suggest reproductive impairment due to anthropogenic noise, which could potentially contribute to decreased crustacean populations and subsequent ecological and economic repercussions. Given the findings of our preliminary study, more research should be undertaken that includes larger sample sizes, double blind setups, and controlled laboratory trials in order to more fully extrapolate the potential impact of noise on mating in the natural environment.

## Introduction

Anthropogenic noise has well established deleterious effects on a range of taxa [1], the most widely studied impacts of anthropogenic noise have been primarily on its direct interference with animal communication [2, 3]. Mate signalling systems have been particularly well studied in birds [4–6] but noise also has the potential to cause impacts even where acoustic communication is not an obvious component of the mating system. Mating behaviours may be particularly vulnerable to disruption by external stimuli because the act of mating can place participants in a more vulnerable position, promoting individual caution in relation to engaging in copulation when there is any source of disturbance [7, 8]. Furthermore, females are likely to choose among males, which may lead to them being particularly sensitive to environmental conditions and may increase rejection rates in sub-optimal contexts. This in turn may select for males that abandon mating attempts unless conditions are optimal [8].

The extent to which anthropogenic noise impacts marine animals has only recently started to be understood in more depth [9–11]. The current literature surrounding anthropogenic sound in the marine environment has primarily focused on vertebrates such as marine mammals and teleost fish [12], with particular attention to cetaceans as they heavily rely on bioacoustics for their daily functioning. Marine invertebrates are also able to detect noise through sound-produced particle motion, and a recent study shows that some crustacean species are sensitive to a combination of particle acceleration and pressure [13]. Many species of marine invertebrates have been shown to be negatively affected by noise generated by anthropogenic activities [14].

Within the limited number of noise studies on marine invertebrates, crustaceans are one of the best represented taxonomic groups [12] with evidence for impacts on survival and function at all stages of development [15]. Larvae from two species of crab were shown to have delayed metamorphosis and decreased settlement due to anthropogenic noise from playback recordings of tidal and offshore wind turbines [16]. Work on shore crabs (*Carcinus maenas*) showed that crabs were more likely to have disrupted foraging behaviour, longer times to retreat to shelter, and longer times to right themselves in the presence of a perceived predator [17]. They were also found to be more likely to suffer physiological stress in the presence of recorded ship noise [18]. Subsequent studies of juvenile shore crabs have shown that playbacks of ship noise inhibited colour change and camouflage, slowed growth, delayed moulting, and impaired antipredator behavioural responses, even compared to treatments with ambient noise of a similar amplitude to the ship noise [19]. In adult crustaceans, anthropogenic noise has been shown to be associated with elevated stress responses [20, 21] and damage to balance organs (statocysts) [22]. Noise has also been shown to disrupt agonistic interactions [23], conspecific grouping [24], and shell selection behaviour [25]. Despite the growing literature on impacts of anthropogenic noise on crustacean behaviour, mating remains understudied. There is some evidence for effects on behaviour impacting aspects of reproduction [26]. For example, dominant male rock shrimp (*Rhynchocinetes typus*) reduce their activity under noise playback conditions, allowing subordinate males to intercept and mate with females [26].

The timing of mate attraction in some decapod crab species is crucial for reproductive success, as copulation is only possible for a short time after a female has moulted and her shell is soft [27]. Females of many species of crabs announce this impending moult to potential mates through a specific chemical cue released in their urine [28]. In the shore crab, this pheromone is uridine diphosphate (UDP) [29] which is a nucleotide accumulated during chitin biosynthesis [30]. If this sex-pheromone is detected by chemoreceptors located on males’ antennules and conditions allow, mating rituals begin until final copulation unless interrupted by competing males [31].

Detecting and responding to chemical cues is a crucial function for animals who use these cues to find food, available shelter, mates, and to detect predators [31, 32]. However, as humans continue to add stimuli to the environment through noise, light, chemical pollution, greenhouse gases and more, this may interfere with an organism’s ability to detect and respond to such cues [33, 34]. Hermit crabs (*Pagurus acadianus)* use a chemical cue to find available gastropod shells for shelter, yet it has been found that after exposure to anthropogenic noise, they are less likely to be attracted to the chemical cue [25]. Since the mating behaviour of shore crabs (and that of numerous other marine crustaceans) also relies on chemical communication, noise has the potential to cause disruption in green shore crabs and other taxa. In contrast to previous studies [35], a recent study measuring shore crabs’ ability to find food using olfactory senses under noise playback revealed no significant effects of noise on a crab’s efficiency, time, or success in finding food [36]. Such contradicting results highlight the need for further research into the effects of noise on chemical perception in crabs.

To test the effects that anthropogenic noise may have on crustacean mating behaviour, we focussed on the shore crab (*Carcinus maenas*). This species is common on UK coastlines, although we have personally observed some population declines off parts of the coast of Cornwall in recent years, and its life history and mating behaviour have been extensively studied. We hypothesized that if the presence of anthropogenic noise can impact acquisition of a valuable resource such as an available shell [25] or food [35] (but see [36]), noise may also inhibit a crab’s ability to respond to a sex-pheromone or cause males to prioritise vigilance over mating behaviour. If anthropogenic noise can disrupt mating behaviour, it has the potential to affect the reproductive success and therefore population density and distribution of a common crustacean species.

## Methods

### Ethical note

All experiments were carried out under the approval (application ID eCORN003542 v3.3) of the University of Exeter’s Biosciences Ethical Committee and adhere to all UK legal guidelines. No crabs were harmed during or after the experiments and all were returned to the places they were collected on the same day. *Carcinus maenas* is not an endangered species and individuals were not removed from the site, therefore no licenses or permits were needed for this study as all work was conducted in locations that were publicly accessible and under ethical approval of the full collection and experimental protocols.

### Collection and setup

**Fig 1:**
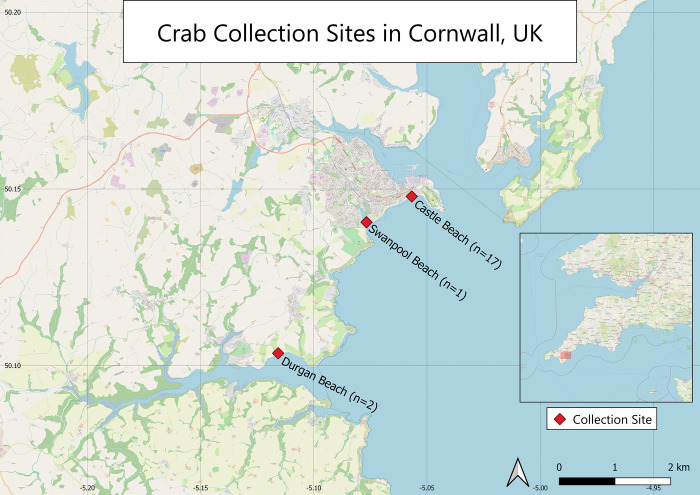
Map of crab collection sites Durgan, Swanpool and Castle beaches located around the southwestern coast of Cornwall with number of crabs caught at each site.

Male crabs (n = 30, 15 each treatment) of at least 30 mm carapace width (range = 32–58 mm) were collected by hand within two hours of low tide from rocky areas in the intertidal zone on beaches located around Falmouth, Cornwall, UK (50.152573, -5.066270) between 16 June and 9 August 2021 (Fig 1).

**Fig 2:**
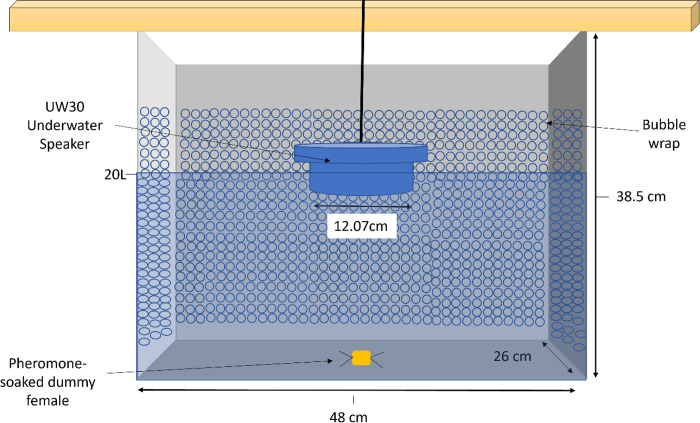
Experimental tank setup with 1) bubble wrap lining three sides of the interior of the tank to reduce sound reverberation, 2) hanging underwater speaker so just speaker is submerged under the water line from a horizontal wooden plank, and 3) pheromone-soaked sponge made to resemble a female crab.

All experiments were performed on the beach immediately after crab collection, in part owing to COVID-19 restrictions limiting access to laboratories for experiments. Experiments were only performed in locations far removed from other anthropogenic activities (such as beachgoers) to limit any external noise during the trials and we did not notice any impact of external noise on trials. A transparent, plastic 35-liter tank (48 × 38.5 × 26 cm) lined with bubble wrap to prevent noise reverberation was filled with 20 litres of seawater collected at the site (Fig 2). Collected crabs were held in covered buckets containing seawater from the upper intertidal zone for no more than 30 minutes before experiments were performed. During acclimation and experimental periods, light blocking material covered the top and three sides of the tank to limit light and reduce stress in the crabs from perceived overhead predation vulnerability. Visual observations were conducted and video recordings were taken when possible.

### Sound treatment

Recordings taken from three ships (a tanker, container ship, and ferry) in UK harbours–see [17]–were edited together into one, 30-minute MP3 file using the sound editing software program Audacity 2.4.2 (www.audacityteam.org). Each ship recording was edited in Audacity to comprise a 30 second fade in, followed by 4 minutes of continuous noise, and a 30 second fade out with one second of silence in between to simulate the noise of a passing ship [17]. Ship recordings were then randomly alternated for the duration of the track to avoid habituation and to mimic the conditions of a busy harbour and each trial began at a randomized section of the recording. This design ensures that crabs in the ship-noise treatment experienced noise from several different ships. The limited number of ship noise recordings that we had access to means that there is an outside possibility that something very unusual about one of the ship-noise recordings might mean that any differences we observe cannot be generalised to ship noise *per se*. However, we have no reason to think that this is the case, and it is much more likely, that if our ship noises are associated with a change in crab behaviour that other anthropogenic noises, including ships, are also likely to disrupt mating behaviours. Additionally, in a similar study on anthropogenic noise on shore crab behaviour, no differences were seen between these three ships using the same recordings (see page 37 in [37]).

**Fig 3:**
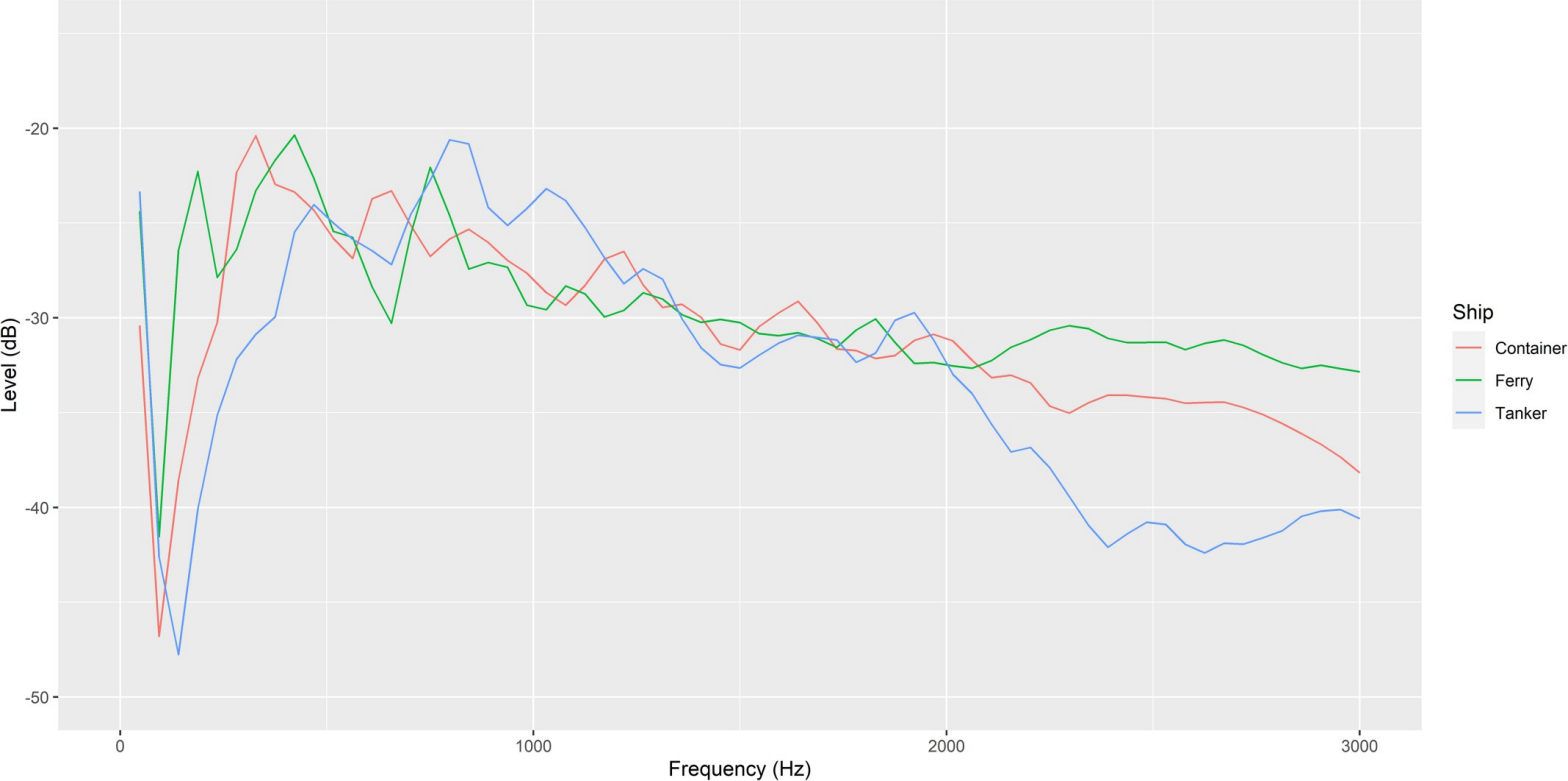
Spectrograph (Hann window, size 1024) showing the average sound profile (amplitude/level and frequency) of the original recording of three ships recorded in UK harbours approximately 200 metres away [17].

**Fig 4:**
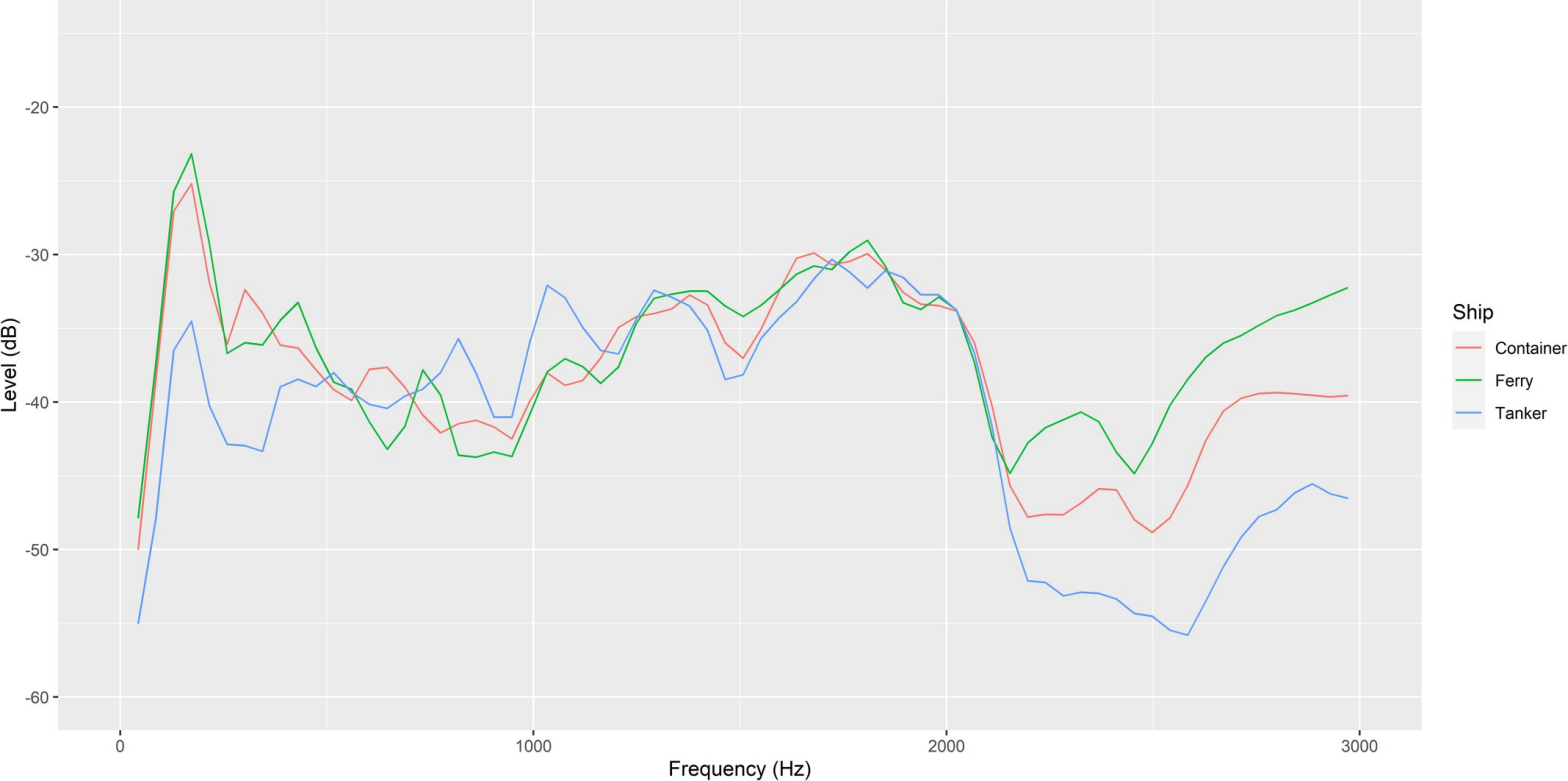
Spectrograph (Hann window, size 1024) showing the average sound profile (amplitude/level and frequency) of the in-tank noise levels of playback recordings [17].

The full recording was played using a suspended UW30 Underwater Loudspeaker (University Sound Diatran Omni-directional Underwater Loudspeaker; frequency response 100–10,000 Hz) with an amplifier (Kemo Electronic; 18W) at a consistent amplitude for each trial. The speaker was hung horizontally in the middle of the tank by a wooden plank so that just the speaker was submerged under the surface of the tank water. 30-second sections from the middle of each recording were analyzed in Audacity through the Plot Spectrum function and plotted in R to show the average sound profile for each ship (Fig 3). Fiddler crabs (*Uca* spp.) and other decapod species are believed to have a frequency sensitivity range between 30–3,000 Hz [38] so anything outside of this range is likely to not be detected by the tested crabs and therefore was not included in the graph [37]. Speakers were powered using a powerbank (Ravpower; 80W; 20,000 mAh) and recording was played back through a Ruizu MP3 player at a consistent amplitude for each trial [37]. In a previous study, the playback of ship noise itself, regardless of amplitude, produced negative responses in shore crabs, so amplitude was not precisely controlled for [19]. Additionally, producing an amplitude within our tank that matches the precise amplitude of a passing ship *in situ* would be highly challenging as it would vary with the distance, type of ship and conditions. The experimental sound playback and ambient noise intensity level was recorded in our tank on the beach during low tide using an Aquarian H2a hydrophone hung 60 mm from the bottom, in the centre of the tank [37]. Sound output was recorded on a Tascam dr-05x recorder. The mean sound intensity and peak level of the original sound recordings, the in-tank sound recordings, and the ambient treatment were recorded and analysed in Audacity using the WaveStats plug-in (Table 1, Fig 4).

**Table 1:** Average sound levels for ambient noise, original recording and in-tank recordings. RMS = root mean squared; A = A-weighted; FS = relative to a full scale where the maximum decibel level is 0.0; Peak = maximum decibel level recorded [37].

Sound Treatment	RMS(A) dBFS	RMS dBFS	Peak dBFS
Ambient	-68.1	-42.4	-22.7
Original Ship 1	-14.9	-13.7	-0.4
Original Ship 2	-15.2	-14.1	0.0
Original Ship 3	-15.1	-13.7	-0.3
In-Tank Ship 1	-21.9	-22.4	-1.4
In-Tank Ship 2	-19.2	-19.7	-1.5
In-Tank Ship 3	-15.7	-15.7	0.0

### Pheromone and dummy female

For the sex-pheromone, a 10^−3^ M solution was mixed using 97.7 mg of uridine 5′-diphospho-N-acetylglucosamine sodium salt (C_17_H_25_N_3_O_17_P_2_Na_2_) (Sigma-Aldrich) in 150 ml of seawater [39]. This solution was then stored in a -20°C freezer in between experimental days and kept cool using a cooler and ice packs in between trials. To simulate a sexually mature female, a yellow, natural (untreated) sponge measuring at least 5 mm less than the width of the test crab’s carapace was soaked in the UDP solution for five minutes prior to the experiment [39]. Two toothpicks were inserted diagonally into the sponge to simulate legs and a small pebble was placed inside the sponge to maintain negative buoyancy [39]. Each sponge was used only once per day and was thoroughly cleaned between experiments using alternating rinses of alcohol, freshwater, and seawater.

### Behavioural assays

Shore crabs display clear, sequential behaviour associated with mating [40]. Both posing and cradle-carry behaviour are distinct and unique to mating, therefore only observations of these behaviours were considered a positive mating response. During behavioural assays, posing was recorded if the crab rose onto the tips of its pereopods with the fifth pereopods raised to at least carapace height and chelipeds extended 90 degrees from the body. Cradle-carry was recorded if the crab grabbed, mounted, and hooked the sponge with one of its walking legs. A shortcoming in our experimental design was that for logistical reasons, the experimenter (KR) was not blind to the treatment in each trial. However, when courtship behaviour occurred it was very distinctive so there would have been very little opportunity for subconscious bias on the part of the observer to influence our results.

For each treatment, the crab was placed into the experimental tank and given five minutes to acclimate. After this period, the dummy-females soaked in the UDP solution were placed into the centre of the tank and the timer started. In both ambient and noise treatments, the speaker was left in the tank to be consistent across treatments. In noise treatments, the noise recording was started immediately when the sponge was introduced to the tank. In both treatments, behaviour was then monitored over 30 minutes, with recordings taken if mating behaviour (posing or cradle-carry) was observed and the time it took from the start of the experiment to when the crab displayed the behaviour. If no response was seen after 30 minutes, the experiment was concluded, and the crabs were returned to the collection site.

### Statistical analysis

All statistical tests were produced using the statistical analysis software RStudio (R v.1.3.1093). To analyze and compare the observed responses in both the no noise and noise conditions, a Bernoulli distribution using a binomial generalized linear model was used, as this is the best for non-parametric, binary data when controlling for other variables. To analyze the response times in both conditions, a gamma family generalized linear model with a two-parameter distribution was used as this is an appropriate test for non-parametric, time-series data when the variance is proportional to the mean squared [41]. Crab size was controlled for, as bigger males may be physically more fit and able to compete for females against smaller males, and therefore may be more likely to respond to a female cue [42]. Time of day was also controlled for, as trials were not conducted at the same time each day and the differences in light levels or other factors may have influenced the response rates of the crabs.

## Results

### Mating responses

Of the 15 crabs that were exposed to ambient noise, 13 (87%) produced a posing mating response, including two crabs that also displayed cradle-carry behaviour with the pheromone-soaked dummy-female. In the noise playback treatment, only six out of 15 crabs (40%) responded, with five showing posing behaviour and one cradling the sponge. A significant, negative effect of noise on the presence of mating responses in male shore crabs was found (Binomial GLM: b ± SE = -2.26 ± 0.928, *X*^2^_28_ = 31.9, P = 0.007, note that the estimate is on a logit scale). Size and time of day were controlled for but were found to be not significant predictors of the mating response (*X*^2^_26_ = 31.0, P = 0.640 and *X*^2^_26_ = 31.0, P = 0.339, respectively).

### Response times

**Fig 5:**
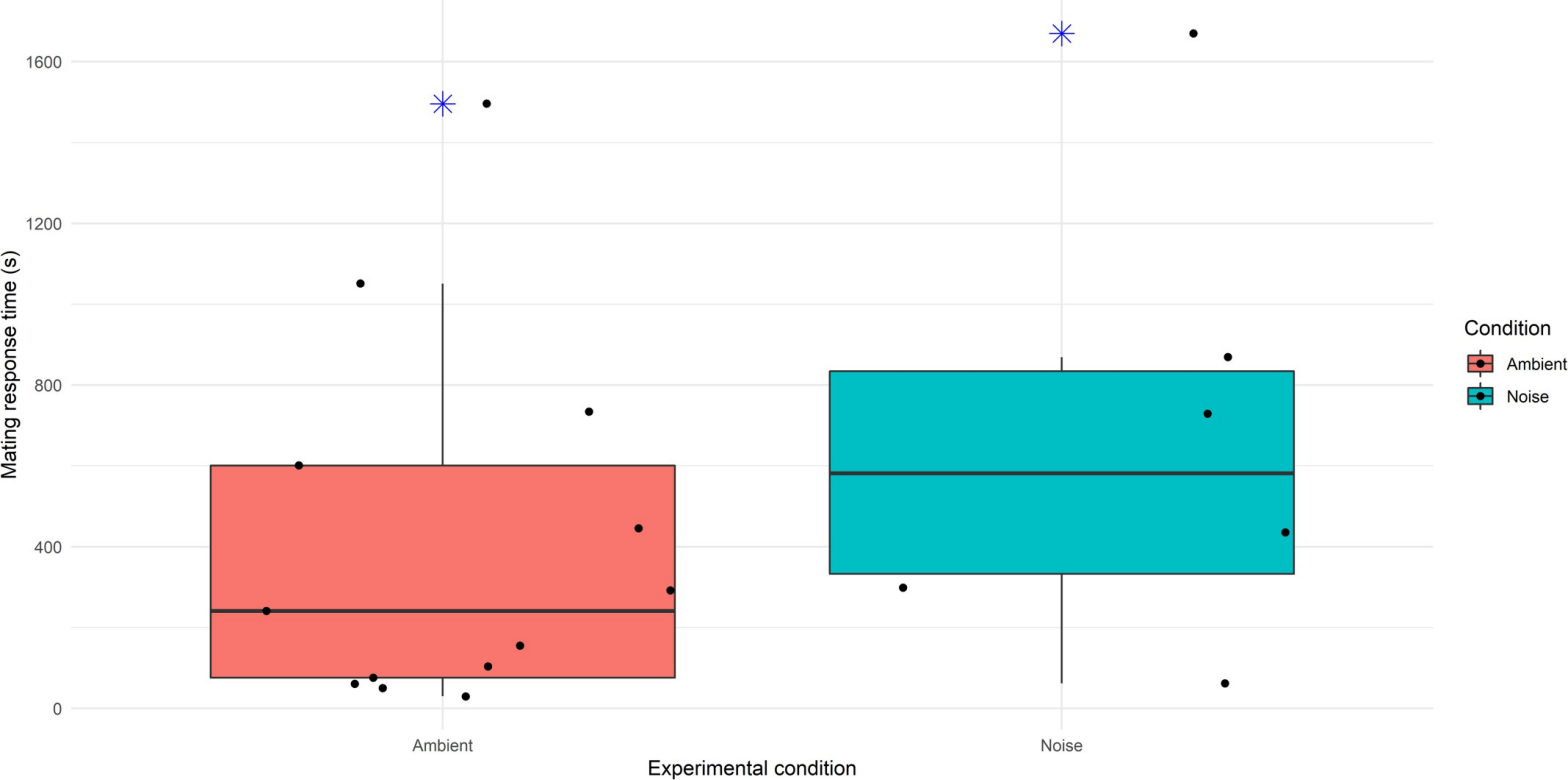
Boxplot comparing the median (horizontal line dividing the boxes), mean (x), range (upper and lower whiskers), 25% (lower box boundary) and 75% quartiles (upper box boundary), outliers (asterisks) and jittered points (dots) for crab mating response times in seconds under noise (n = 6) and ambient conditions (n = 13).

For individuals that showed a mating response, the mean time it took for crabs in the ambient treatment to respond was 410 seconds (n = 13), compared with 678 seconds in the noise playback treatment (n = 6) (Fig 5). The results showed some directionality, however there was not a significant effect of noise on the time it took for crabs to produce a response (Gamma GLM: b ± SE = -6.58e^-04^ ± 10.0e^-04^, F_16_ = 0.424, P = 0.524). Again, both size and time of day did not have a significant effect on mating response times (F_15_ = 0.530, P = 0.478 and F_15_ = 1.924, P = 0.186, respectively).

## Discussion

We found that exposure to anthropogenic noise had a significant, negative effect on the presence of mating behaviour (posing and cradle-carry) in adult, male shore crabs when presented with the synthetic sex-pheromone UDP. Crabs that were exposed to an experimental playback recording of ships were less likely to respond to a dummy-female soaked in UDP in posing or cradle-carry mating behaviour. Noise in this study, however, did not cause a significant difference to the time it took crabs to respond to the pheromone, which could be a result of small sample sizes, as crab mating response times showed a slight, albeit insignificant, direction towards being slower in the presence of noise. To our knowledge, this is the first study examining the effects of noise on crab mating and the first indication that noise may interrupt such vital reproductive behaviour in this species. Our findings add to the limited existing literature that indicate noise does indeed impact mating behaviour in other marine species [43].

At present we can only speculate about the mechanism by which noise affects male mating behaviour. Noise could distract the crabs, leading them to redirect their attention away from the chemical cues they receive leading them to fail to respond to the sex-pheromone. Previous studies have suggested distraction as the cause to behavioural changes observed under noise conditions when the masking effect of noise was controlled, as seen in Caribbean hermit crabs (*Coenobita clypeatus)* [44]. In this study, hermit crabs allowed a simulated predator to approach more closely under noise conditions, regardless of whether the simulated predator produced noise or not [44]. Crabs could also be perceiving noise as a threat itself, in which failing to respond to a mating cue may represent the crab’s ability to weigh the costs and benefits of pursuing a potential mate under life-threatening scenarios. Such decision-making abilities of crustaceans when exposed to noise has been suggested by researchers who found noise to reduce a crustacean’s attraction to the chemical cue for an available resource [25]. Noise may also simply represent a chronic stress that increases allostatic load, or the cumulative strain chronic and repeated stress has on the body, and thereby reduces the capacity of the crab to carry out a range of functions including the mating behaviours that we examined [45, 46]. In one study, shore crabs consumed 67% more oxygen following singular exposure to ship noise compared to those exposed to ambient noise, and oxygen consumption stayed elevated across repeated ship noise playback suggesting the potential for noise to cause chronic metabolic stress [18]. Alternatively, while crab size was not found to be a significant factor in response rates or times, we were not able to control for previous competition/fighting amongst males, which may affect the response rates that were observed as any male that had recently lost in competition may not have responded or may have had a delayed response to the pheromone [42].

If male crabs are less likely to respond to mating cues under noise conditions due to distraction, predator avoidance, or elevated stress, this would ultimately lead to a reduction in the number of partners available to sexually receptive females. The extent to which female shore crabs are mate-limited in nature is unknown but given the timing constraints that their moult places on mating [27], it is possible that some females might fail to fertilise their eggs if males are adversely affected by anthropogenic noise. Mating delays in post-moulted females have been shown to significantly affect fertilization rates and reduce the number of surviving offspring in female spiny king crabs (*Paralithodes brevipes*), where there is an inverse relationship between the number of days from moulting to mating and egg survival [47]. Without successful recruitment of larvae into the population, cascading negative effects on species densities and distributions are inevitable. Additionally, the negative effects of noise on crustacean behaviour may even be compounded with multiple anthropogenic stressors such as ocean acidification, pollution, and climate change, therefore contributing to decreasing populations [34, 48, 49]. Shore crabs are known to be a highly adaptable and robust species, traditionally one of the most common crabs around the UK coast, yet in our personal observations we have noted a substantial decline in their abundance in the last decade at our study sites in Cornwall. The possible reasons are speculative, yet it is possible human impacts are at least contributing to this.

Our results have wider implications for coastal and marine biodiversity, ecosystem functioning, commercial fisheries, and pest management. Green shore crabs represent only one species of crustacean that use pheromones to locate resources and available mates; many other species show similar behaviour and may be impacted in comparable ways [39]. Crustaceans provide vital ecosystem services; they link benthic and pelagic species in the trophic system, decompose organic materials, and use filter feeding to maintain water quality [50]. They are also economically important to global fisheries: 7% of the world’s total catch weight in 2018 was of crustaceans alone [51]. Without mitigation, decreasing populations in both commercially important and nontarget crustacean species could upset the delicate balance of the ecosystem, the food web, and contribute to the collapse of major commercial fisheries. Contrarily, population effects to shore crabs may be a welcomed consequence by marine environmental managers. Shore crabs are an invasive species in several countries around the world [52], therefore any understanding of mechanisms which may impact their populations could be seen as valuable for invasive species eradication.

It is important to note that these are preliminary findings and further research should be done with greater sample sizes, double blind setups and both real ship noise and female crabs. Because a playback of ship noise was used (instead of the noise of a real ship), we can only report the effect of general anthropogenic noise on mating behaviour in response to a dummy-female soaked in synthetic sex-pheromone, and caution is needed in extrapolating specifically to the effect of *in situ* ship noise itself on mating. In future studies it would be interesting to control for temperature and pH of the water and also conduct this experiment within a laboratory setting, as crabs that have been acclimated to a laboratory environment may produce different results due to lowered initial stress levels. Measuring the effect of anthropogenic noise at different times during the mating response may also be an interesting future avenue for research, as crabs that perceive noise as a threat may continue to guard the female as a protective response or may become distracted and release the female during cradle-carry.

## Supporting information

S1 Data (XLSX)
